# Semi-supervised multi-label classification using an extended graph-based manifold regularization

**DOI:** 10.1007/s40747-021-00611-7

**Published:** 2022-01-04

**Authors:** Ding Li, Scott Dick

**Affiliations:** Department of Electrical and Computer Engineering, University of Alberta, Edmonton, AB Canada T6G 1H9

**Keywords:** Manifold regularization, Multi-label classification, Semi-supervised learning, Graph-based learning

## Abstract

Graph-based algorithms are known to be effective approaches to semi-supervised learning. However, there has been relatively little work on extending these algorithms to the multi-label classification case. We derive an extension of the Manifold Regularization algorithm to multi-label classification, which is significantly simpler than the general Vector Manifold Regularization approach. We then augment our algorithm with a weighting strategy to allow differential influence on a model between instances having ground-truth vs. induced labels. Experiments on four benchmark multi-label data sets show that the resulting algorithm performs better overall compared to the existing semi-supervised multi-label classification algorithms at various levels of label sparsity. Comparisons with state-of-the-art supervised multi-label approaches (which of course are fully labeled) also show that our algorithm outperforms all of them even with a substantial number of unlabeled examples.

## Introduction

In many real-world applications, such as bioinformatics and video annotation, obtaining labeled data is sometimes very difficult, expensive and time-consuming. On the other hand, it may be simple and inexpensive to obtain unlabeled data. For instance, vast numbers of videos and images are available on the web. The large amount of unlabeled data can reveal useful information about the phenomena we are studying, e.g., estimating the distribution of the data as well as the data structure [68]. As a result, Semi-Supervised Learning (SSL) is drawing increasing interest in the machine-learning community [10].

Studies on SSL are extensive (e.g. [2, 4, 12, 13, 32, 45, 51, 62, 66]); detailed reviews may be found in [65] and [42]. The common purpose of semi-supervised algorithms is to exploit both labeled data and unlabeled data to create superior classifiers compared to labeled data alone. According to [10], self-training (also known as self-learning or self-labeling) is among the earliest approaches that use unlabeled data in classification. The idea of the self-training first appeared in [41]. In self-training, a classifier is first trained only with the labeled data, and then used to predict labels for some unlabeled data. Then, the classifier is re-trained with both the ground-truth and predicted labels, and used to predict additional labels. The process repeats until all examples are labeled. The authors in [42] use the expectation-maximization (EM) algorithm [14] for SSL. Co-Training [6] is a learning paradigm to address problems with strong structural prior knowledge available, and is regarded as a variant of EM on the probabilistic model [10, 42]. It assumes that features can be split into two complementary and independent feature subsets and each feature subset is enough to train a classifier for the data. Then, each classifier uses its most confidently predicted points and their labels to teach the other classifier. The process of using the other classifier’s most confidently predicted labels to teach itself is iterated until some criteria is achieved. Transductive learning is another approach, based on the idea of performing predictions only for test samples [10]; Transductive Support Vector Machines (TSVM) are one example [54]. Various extensions to the TSVM have been proposed [9, 11, 16, 60]; the common point is that the algorithms try to learn a hyperplane over the labeled data and the unlabeled data by optimizing a tradeoff between maximizing the margin over the labeled data and regularizing the decision boundary over low-density regions of all data samples.

Graph-based algorithms are an important sub-class of SSL that have recently attracted considerable attention [10, 48, 49]. Various graph-based SSL algorithms have been developed [3, 5, 25, 28, 53, 55, 56, 59, 64, 67] and a number of successful applications can be found in recent publications [1, 29, 30, 61]. Some popular graph-based algorithms include Local and Global Consistency [64], Gaussian Random Fields and Harmonic Functions [67], mincuts [5], greedy max-cut [55], and spectral graph transducers [28]. All the graph-based algorithms begin by constructing a graph with nodes representing data points, and edges representing similarity between the connected nodes. The labeled data points are then used to perform graph clustering or propagate labels from labeled points to unlabeled points, by minimizing the empirical cost over labeled data and regularizing the smoothness over the graph using all the data. Another representative SSL approach is manifold regularization [3], which assumes data points lie on a low-dimensional manifold in the input space [20, 35, 50].

At the same time, most above semi-supervised classification algorithms implicitly assume that class labels are mutually exclusive. However, in many application domains, such as image classification, bioinformatics and news categorization, each instance can represent more than one concept simultaneously; this is best represented as a vector of labels. In addition, human emotions and sentiments are sometimes regarded as a multi-label classification problem nowadays, e.g., multiple fine-grained emotions may coexist in a single tweet of a microblog [21]. In addition, multi-label classifiers have recently been utilized for recognizing crop diseases in agriculture [27]. The learning algorithms for these problems are the “multi-label classifiers” as reviewed in [47, 58]. For instance, a well-known multi-label classifier is the Multi-Label k Nearest Neighbors (MLkNN) [57], which is an extension of the classical kNN method. References [31, 37], and [39] study a variety of supervised multi-label algorithms and present extensive experiments to compare their performances.

Our focus in the current paper is the intersection of these two problems, to wit, the design of semi-supervised multi-label classifiers. There is relatively less work in the literature on this sub-problem, and a particular dearth of graph-based semi-supervised algorithms for the multi-label case. Some existing studies on semi-supervised algorithms include the Multi-Label Gaussian Fields and Harmonic Functions (ML-GFHF) [56], the Multi-Label Local and Global Consistency (ML-LGC) [56], the Fixed-Size Multi-Label Regularized Kernel Spectral Clustering (ML-FSKSC) [33], and the Semi-Supervised Weak-Label approach (SSWL) [18]. In spite of these results, the opportunities in this area are extensive. Better methods are needed for semi-supervised multi-label classification in many tasks.

In our previous work [29], we found that a multi-label extension of the Manifold Regularization algorithm [3] was quite effective for non-intrusive load monitoring. In the current paper, we seek to improve upon that algorithm, and determine how well our results generalize beyond that domain. We investigate a multi-label extension of the Manifold Regularization (MR) algorithm, augmented with a reliance weighting strategy to further improve classification performance. Reliance weights allow learning algorithms to differentiate between ground-truth and induced labels in constructing a classifier for a given data set. They take the form of an additional matrix term in the kernel expansion of the Laplacian Regularized Least Squares model learned in MR [3]. We evaluate our proposed algorithm in comparison with five other multi-label algorithms (four semi-supervised algorithms plus MLkNN), on a set of four benchmark data sets.

The key contributions of this work are:

- The manifold regularization algorithm is extended to learn multi-label classifiers.
- A weighting strategy is proposed to vary the trust placed in labeled and unlabeled instances when forecasting labels for unseen points.
- The proposed approach is compared against four semi-supervised, and one fully supervised, multi-label algorithms, and performs as well as or better than all of them.

The advantages of the proposed method are threefold: (1) the proposed method performs as well or better than the existing semi-supervised multi-label algorithms on the four data sets in the fifth section. It furthermore outperforms the state-of-the art supervised multi-label algorithms (which of course are trained on fully labeled data), even when a substantial portion of the training set is unlabeled. (2) The proposed method has a low model complexity as the Manifold Regularization [3] assumes data points lie on a low-dimensional manifold in the input space. (3) The proposed reliance weighting strategy allows an analyst to specify different trust levels for ground-truth and induced labels. The disadvantage of the method mainly lies in the computational time required for the construction of the graph structure; this is a common problem in this class of algorithms.

The remainder of this paper is organized as follows: the next section presents the preliminaries, including introducing the basis and notations, regularization in reproducing Kernel Hilbert space and manifold regularization. The third section presents the proposed approach, including graph construction, manifold regularization with multiple labels and our reliance weighting strategy. The fourth section describes the experimental design including introducing the data sets, experimental setup, performance metrics and statistical significance tests. The fifth section presents our experimental results and discussion, and we offer a summary and discussion of future work in the last section.

## Preliminaries

This section presents the notations and basics that are used throughout the paper, and reviews the manifold regularization algorithm.

### Basics and notations

In the framework of semi-supervised learning, the data set $\mathbb {D}$ in the training phase consists of two parts, namely $\mathbb {D}=\mathbb {D}_l\cup \mathbb {D}_u$, where $\mathbb {D}_l$ and $\mathbb {D}_u$ indicate the labeled and unlabeled training data sets, respectively. Both $\mathbb {D}_l$ and $\mathbb {D}_u$ are drawn from the same distribution $p(\mathbf {x})$, where $\mathbf {x}$ indicates a feature variable. In the single label case, the feature space and label space of a data set $\mathbb {D}$ are denoted by $\mathcal {X}=\mathbb {R}^d$ and $\mathcal {Y}=\{-1,1\}$, respectively. Then, the labeled and unlabeled training data sets are represented by $\mathbb {D}_l=\{(\mathbf {x}_i,y_i):\mathbf {x}_i\in \mathcal {X},y_i\in \mathcal {Y}, i=1,2,\ldots ,l\}$ and $\mathbb {D}_u=\{\mathbf {x}_i: \mathbf {x}_i\in \mathcal {X}, i=l+1,l+2,\ldots ,l+u\}$, where *l* and *u* indicate the numbers of labeled and unlabeled instances $\mathbf {x}_i=[x_{i1},x_{i2},\ldots ,x_{id}]^T$ for $i=1,2,\ldots ,n$, where *d* indicates the feature dimension. The total number of all training instances in $\mathbb {D}$ is $n=l+u$. The goal of semi-supervised learning with single label is to infer the labels ${\tilde{Y}}=\{{\tilde{y}}_i\in \mathcal {Y},i=1,2,\ldots ,e\}$ for future instances $\mathbb {D}_e= \{{{\tilde{\mathbf{x}}}}_i \in \mathcal {X},i=1,2,\ldots ,e\}$ given the training data set $\mathbb {D}=\mathbb {D}_l\cup \mathbb {D}_u$. [49, 68]

In the multi-label case, the label space of $\mathbb {D}$ is denoted by $\mathcal {Y}=\{-1,1\}^L$, where *L* indicates the number of labels. Analogously, the labeled training data set becomes $\mathbb {D}_l= \{(\mathbf {x}_i, \mathbf {y}_i): \mathbf {x}_i\in \mathcal {X},\mathbf {y}_i\in \mathcal {Y}, i=1,2,\ldots ,l\}$ and the label vector is $\mathbf {y}_i=[y_{i1},y_{i2},\ldots ,y_{iL}]^T$, whereas the other notations remain the same as the single label case. The goal of semi-supervised learning with multiple labels is to infer the labels ${{\tilde{\mathbf{Y}}}}=\{{{\tilde{\mathbf{y}}}}_i\in \mathcal {Y},i=1,2,\ldots ,e\}$ for $\mathbb {D}_e=\{{{\tilde{\mathbf{x}}}}_i\in \mathcal {X},i=1,2,\ldots ,e\}$ given $\mathbb {D}=\mathbb {D}_l\cup \mathbb {D}_u$.

Using the graph-based semi-supervised learning, a crucial step is to construct a graph $\mathcal {G}=(V,E)$ representing the connections between training instances $\mathbf {x}_i\in \mathcal {X}$ [49, 56, 68]. Specifically, $\mathcal {G}=(V,E)$ has *n* vertices $V_i$ and each vertex $V_i$ represents an instance $\mathbf {x}_i,i=1,2,\ldots ,n$. $E_{ij}$ is an edge connecting vertices $V_i$ and $V_j$. There are three typical methods to construct such a graph, including the *k* nearest neighbor algorithm, $\varepsilon$ distance measure and full connection. For example, using the *k* nearest neighbor algorithm, each edge $E_{ij}$ connects the vertices $V_i$ and $V_j$ if vertex $V_i$ is among the *k* nearest neighbors of vertex $V_j$, or vertex $V_j$ is among the *k* nearest neighbors of vertex $V_i$. A weight matrix $\mathbf {W}$ is defined over the graph $\mathcal {G}=(V,E)$, where $W_{ij}$ is the weight associates with edge $E_{ij}$ representing the similarity between vertices $V_i$ and $V_j$ (namely the training instances $\mathbf {x}_i$ and $\mathbf {x}_j$). Then, the unnormalized graph Laplacian is given by $\mathbf {L} = \mathbf {D}-\mathbf {W}$, where $\mathbf {D}$ is a diagonal matrix with $D_{ii}=\sum _{j=1}^{N} W_{ij}$.

The label inference in graph-based SSL is usually based on two graph assumptions [56, 68]: (1) the prediction should be close to the given labels on labeled vertices; (2) the prediction should be smooth on the whole graph (i.e., vertices that are close in the graph tend to have the same labels). The label inference algorithms for graph-based SSL can be categorized into two major classes: transductive learning (e.g., the graph Laplacian regularization [64, 67]), and inductive learning (e.g., the manifold regularization [3]). Transductive learning infers labels only on the unlabeled training data and cannot make predictions on out-of-sample data. By contrast, inductive learning infers labels for the whole domain, i.e., a function $f:\mathcal {X}\rightarrow \mathcal {Y}$ is learned given $\mathbb {D}=\mathbb {D}_l\cup \mathbb {D}_u$ and then the labels for $\mathbb {D}_e$ are predicted. The work in this paper is based on the manifold regularization [3], which is a typical inductive learning method [63]. The next subsection revisits regularization in a reproducing kernel Hilbert space, which is the core of manifold regularization.

### Regularization in reproducing kernel Hilbert space

For a Mercer kernel $K:\mathcal {X}\times \mathcal {X} \rightarrow \mathbb {R}$, there exists an associated Reproducing Kernel Hilbert Space (RKHS) $\mathcal {H}_K$ of functions $\mathcal {X} \rightarrow \mathbb {R}$ with the norm $||\cdot ||_K$ [40]. The standard supervised learning estimates an unknown function $f\in \mathcal {H}_K$ from the labeled data set $\mathbb {D}_l$ as

1 $$\begin{aligned} f^*=\mathop {\mathrm{arg\,min}}\limits _{f\in \mathcal {H}_K }\frac{1}{l}\sum _{i=1}^{l}V(\mathbf {x}_i,y_i,f)+\gamma _A ||f||_K^2, \end{aligned}$$

where $V(\mathbf {x}_i,y_i,f)$ is the loss function, such as the squared error loss $(y_i-f(\mathbf {x}_i))^2$ for regularized least squares (RLS). $||f||_K^2$ is a regularization term in the RKHS imposing the smoothness condition on possible solutions. $\gamma _A$ balances the tradeoff between the empirical cost and the regularization term. *l* is the number of labeled instances.

The difference between semi-supervised learning to supervised learning lies in the utilization of the marginal distribution of $\mathbb {D}=\mathbb {D}_l\cup \mathbb {D}_u$ to improve the learning performance in addition to the empirical cost obtained over the labeled data set $\mathbb {D}_l$. According to the discussions in [3], there is an identifiable relation between marginal distribution $p(\mathbf {x})$ and conditional distribution $p(y|\mathbf {x})$, i.e., if two instances $\mathbf {x}_i,\mathbf {x}_j\in \mathcal {X}$ are close in the intrinsic geometry of $p(\mathbf {x})$, then their conditional distributions $p(y|\mathbf {x}_i)$ and $p(y|\mathbf {x}_j)$ are similar. Thus, another regularization term can be added to ensure that the solution is smooth with respect to the marginal distribution $p(\mathbf {x})$. Incorporating the smoothness penalty term with respect to the graph Laplacian $\mathbf {L}$, we derive the following optimization problem [3]:

2 $$\begin{aligned} f^* =\mathop {\mathrm{arg\,min}}\limits _{f\in \mathcal {H}_K }\frac{1}{l}\sum _{i=1}^{l}V(\mathbf {x}_i,y_i,f)+\gamma _A ||f||_K^2+\frac{\gamma _I}{n^2}\mathbf {f}^T\mathbf {L}\mathbf {f},\nonumber \\ \end{aligned}$$

where $\mathbf {f}=[f(\mathbf {x}_1),f(\mathbf {x}_2),\cdots ,f(\mathbf {x}_n)]^T$, and $\mathbf {f}^T\mathbf {L}\mathbf {f}$ is a penalty term that reflect the intrinsic structure of the probability distribution $p(\mathbf {x})$. $n=u+l$ is the number of total instances. The normalizing coefficient $\frac{1}{n^2}$ is the natural scale factor for the empirical estimate of the Laplace operator. Coefficients $\gamma _A$ and $\gamma _I$ controls the complexity of the function in the ambient space and the intrinsic geometry of the $p(\mathbf {x})$ respectively. In real-world data sets, $p(\mathbf {x})$ is unknown, but an empirical estimate can be obtained from a sufficiently large amount of unlabeled data $\mathbb {D}_u$ by assuming the data set lies on a manifold in $\mathbb {R}^d$ and modeling the manifold with the adjacency graph $\mathcal {G}=(V,E)$ from the data set $\mathbb {D}$. According to the classical Representer Theorem [40], the solution to Eq. (2) in $\mathcal {H}_K$ is given by Ref. [3]

3 $$\begin{aligned} f^*(\mathbf {x})=\sum _{i=1}^{l+u}\theta _i K(\mathbf {x}_i,\mathbf {x}), \end{aligned}$$

which is an expansion of the Representer Theorem in terms of labeled data and unlabeled data $\mathbb {D}=\mathbb {D}_l\cup \mathbb {D}_u$. Accordingly, the problem is essentially an optimization problem over the space of coefficients $\theta _i$.

The RKHS has been extended to vector-valued functions [8] to formulate the vector-valued manifold regularization [35]. Let $\mathbf {F} = (f_1(\mathbf {x}_1),\cdots ,f_n(\mathbf {x}_n)) \in \mathcal {Y}^n$ be components of a vector-valued function where each $f_i \in H_K$ [35]. Here $\mathcal {Y}$ can be $\mathbb {R}$ for the single label case or $\mathbb {R}^L$ for multi-label case. The optimization problem of the vector-valued manifold regularization is given by Ref. [35]

4 $$\begin{aligned} f^*= & {} \mathop {\mathrm{arg\,min}}\limits _{f\in \mathcal {H}_K } \frac{1}{l}\sum _{i=1}^{l}V(\mathbf {x}_i,\mathbf {y}_i,f) + \gamma _A ||f||_K^2 \nonumber \\&+ \gamma _I <\mathbf {F},M\mathbf {F}>_{\mathcal {Y}^n}, \end{aligned}$$

where the matrix *M* is a symmetric, positive operator, such that $<y,My>_{\mathcal {Y}^n}$ for all $y\in \mathcal {Y}^n$. $\mathcal {Y}^n$ is the *n*-direct product of $\mathcal {Y}$, with the inner product

$$\begin{aligned}<(y_1,\cdots ,y_n),(w_1,\ldots ,w_n)>_{\mathcal {Y}^n} = \sum _{i=1}^n<y_i,w_i>_{\mathcal {Y}}. \end{aligned}$$

It has been proved in [35] that the minimization problem in (4) has a unique solution taking the form $f^*(\mathbf {x})=\sum _{i=1}^{l+u}K(\mathbf {x}_i,\mathbf {x})\varvec{\Theta }_i$ for some vectors $\varvec{\Theta }_i\in \mathcal {Y}, 1\le i \le n$. The vector-valued manifold regularization is a generalized form of manifold regularization, and can be used for single label, multi-label, and multi-view learning [35, 36].

The Representer Theorem in the vector-valued RKHS is given and proved in [35]. Let $\mathcal {H}_{K,\mathbf {x}} = \{\sum _{i=1}^{u+l}K(\mathbf {x}_i,\mathbf {x})y_i, \mathbf {y}\in \mathcal {Y}^{u+l}\}$. For $f\in \mathcal {H}_{K,\mathbf {x}}^\bot$, the sampling operator $S_{\mathbf {x}}$ satisfies $<S_\mathbf {x}f, \mathbf {y}>_{\mathcal {Y}^{u+l}} = <f,\sum _{i=1}^{u+l}K(\mathbf {x}_i,\mathbf {x})y_i>_{\mathcal {H}_K}=0$. This holds true for all $\mathbf {y} \in \mathcal {Y}^{u+l}$ and yields $S_\mathbf {x}f=(f(\mathbf {x}_1),\ldots , f(\mathbf {x}_{u+l}))=0$. Denote the right-hand side of (4) by *I*(*f*). Any arbitrary $f\in \mathcal {H}_K$, can be decomposed orthogonally as $f=f_0+f_1$, with $f_0\in \mathcal {H}_{K,\mathbf {x}}$ and $f_1 \in \mathcal {H}_{K,\mathbf {x}}^\bot$. This results in $I(f)=I(f_0+f_1)\ge I(f_0)$ with equality if and only if $||f_1||_{\mathcal {H}_K}=0$, since $||f_0+f_1||_{\mathcal {H}_K}=||f_0||_{\mathcal {H}_K}+||f_1||_{\mathcal {H}_K}$. As a result, the minimizer of (4) must lie in $\mathcal {H}_{K,\mathbf {x}}$.

## The proposed method

The work in [3] initially proposed the manifold regularization, and showed that the Representer Theorem minimizes the error for Laplacian RLS in univariate cases; further, reference [35] proved the Representer Theorem for the general cases of the vector manifold regression. Following the two fundamental theoretical works, this work on multi-label manifold regularization is essentially an important special case of the theorem in [35]. In the existing literature, there is no study on such a special case; in particular, no simpler proof has been advanced that the kernel coefficients in Eq. (3) remain a solution to the Laplacian RLS minimization. We are following a long tradition in mathematics where simpler proofs for interesting special cases remain valuable, even if the general case has been proven. For instance, Dirichlet’s theorem was first proved in [17] in the 19th century. Nonetheless, studies of special cases of Dirichlet’s theorem, especially those having elementary proofs (e.g., [24, 38, 43]), continue to this day [34]. Analogously, studying the multi-label classification case of MR also seems an interesting and novel contribution. We also introduce the reliance weighting strategy, and prove that our modified algorithm remains a solution to the Laplacian RLS problem. The major challenges include: (1) the formulation of the optimization problem of manifold regularization with multiple labels given that the data structure is different from the single-labeled data, (2) the solving of the optimization problem to guarantee that a unique global solution exists, (3) the derivation of the solution by including a reliance weight matrix.

### Graph construction

Given the whole data set $\mathbb {D}=\mathbb {D}_l\cup \mathbb {D}_u$, a full $n\times n$ distance matrix $\mathbf {U}$ is calculated between each pair of instances $\mathbf {x}_i, \mathbf {x}_j\in \mathcal {X}$ based on a Gaussian kernel $K(\mathbf {x}_i, \mathbf {x}_j)$ as

5 $$\begin{aligned} U_{ij} = K(\mathbf {x}_i, \mathbf {x}_j) = \exp \left( -\frac{|| \mathbf {x}_i -\mathbf {x}_j ||^2}{2\sigma ^2} \right) , \end{aligned}$$

where $\sigma$ denotes the bandwidth of the Gaussian kernel. Equivalently, an alternative distance matrix $\mathbf {H}$ can be calculated with each element $H_{ij}$ given by Refs. [26, 55]

6 $$\begin{aligned} H_{ij}=\sqrt{U_{ii}+U_{jj}-2U_{ij}}. \end{aligned}$$

The constructed graph $\mathcal {G}=(V,E)$ is a fully connected graph with each edge $E_{ij}$ weighted by $H_{ij}$. According to [26, 55], graph sparsification can improve the efficiency of label inference. Edges are removed producing an $n\times n$ binary matrix $\mathbf {B}$ with 1’s and 0’s representing the presence and absence of connections, respectively. Three sparsification approaches can be used, including the $\varepsilon$-neighbor search, *k*-nearest neighbor search, and the b-matching [26, 55]:

1. The $\varepsilon$-neighbor search recovers a binary matrix $\mathbf {B}$ as
  7 $$\begin{aligned} B_{ij} = \left\{ \begin{array}{ccl} 1 &{} \text{ if } &{} 1-H_{ij}\le \varepsilon \\ 0 &{} \text{ if } &{} 1-H_{ij}> \varepsilon \text{ or } i=j \end{array}\right. . \end{aligned}$$
2. The *k*-nearest neighbor search obtains the binary matrix $\mathbf {B}$ by minimizing the following optimization problem:
  8 $$\begin{aligned} \begin{aligned}&\min _{\mathbf {B}\in \{0,1\}^{n\times n}} \sum _{i=1}^{n}\sum _{j=1}^{n} B_{ij} H_{ij} \\&\text {s.t. } \sum _{j=1}^{n}B_{ij}=k,B_{ii}=0,\forall i,j=1,\ldots ,n. \end{aligned} \end{aligned}$$
3. Using the b-matching algorithm, the optimization problem to recover $\mathbf {B}$ is
  9 $$\begin{aligned} \begin{aligned}&\min _{\mathbf {B}\in \{0,1\}^{n\times n}} \sum _{i=1}^{n}\sum _{j=1}^{n} B_{ij} H_{ij} \\&\text {s.t. } \sum _{j=1}^{N}B_{ij}=b,B_{ii}=0,B_{ij}=B_{ji},\forall i,j=1,\ldots ,n. \end{aligned}\nonumber \\ \end{aligned}$$

The binary matrix $\mathbf {B}$ obtained using the k-nearest neighbor search is not symmetric; thus the final $\mathbf {B}$ can be calculated as $B_{ij}=\max (B_{ij},B_{ji})$. By contrast, the b-matching algorithm produces a graph with every node having the same number of neighbors, namely $\mathbf {B}=\mathbf {B}^T$. Whichever of the above methods is applied, the weight for edge $E_{ij}$ is set to 0 if $B_{ij}=0$. For an edge $E_{ij}$ with $B_{ij}=1$, the weight $W_{ij}$ can be calculated with respect to the distance matrix $\mathbf {H}$ and expressed as

10 $$\begin{aligned} W_{ij}=H_{ij}B_{ij}. \end{aligned}$$

The final graph $\mathcal {G}=(V,E)$ is then constructed and represented by a sparse weight matrix $\mathbf {W}$. Proceeding to label inference, the graph Laplacian is calculated as $\mathbf {L} = \mathbf {D}-\mathbf {W}$, where each element of $\mathbf {D}$ is $D_{ii}=\sum _{j=1}^{N} W_{ij}$ and $D_{ij}=0$.

### Manifold regularization with multiple labels

In this subsection, we extend the manifold regularization in [3] to solve multi-label learning problems. Let $\mathbf {X}=[\mathbf {x}_1,\mathbf {x}_2,\ldots , \mathbf {x}_n]^T$ and $\mathbf {Y}=[\mathbf {y}_1,\mathbf {y}_2,\ldots ,\mathbf {y}_n]^T$ denote the matrix of all feature instances and label instance. In $\mathbf {Y}$, $\mathbf {y}_i$ for $i\le l$ takes 1 or $-1$ for its elements and $\mathbf {y}_i$ is an all-zero vector for $l<i\le n$. In the framework of the Laplacian Regularized Least Squares (LapRLS) [3], the optimization problem of manifold regularization with multiple labels is

11 $$\begin{aligned} f^*= & {} \mathop {\mathrm{arg\,min}}\limits _{f_j\in \mathcal {H}_K,j=1,\ldots ,L } \frac{1}{l} {\text {tr}} \left( (\varvec{\Psi } \mathbf {F}-\mathbf {Y})^T (\varvec{\Psi } \mathbf {F}-\mathbf {Y}) \right) \nonumber \\&+ \gamma _A ||f||_K^2 + \frac{\gamma _I}{n^2} {\text {tr}} \left( \mathbf {F}^T\mathbf {L}\mathbf {F} \right) , \end{aligned}$$

where $\mathbf {F}=[f_j(\mathbf {x}_i)]_{n\times L}, i=1,\ldots ,n, j=1,\ldots , L$ is a matrix representing the predicted outputs, ${\text {tr}}(\cdot )$ denotes the trace of a matrix, and $\varvec{\Psi }$ is a $n\times n$ diagonal matrix with the diagonal elements given by

12 $$\begin{aligned} \Psi _{ii}=\left\{ \begin{array}{ccl} 1 &{} \text{ for } &{} i \le l, \\ 0 &{} \text{ for } &{} l < i \le n. \end{array}\right. . \end{aligned}$$

The second term $||f||_K^2 = \sum _{j=1}^{L}||f_j||_K^2$ in Eq. (11) measures the complexity of $\mathbf {F}$ in the ambient space. The third term represents the intrinsic smoothness with respect to the geometric distribution. $\mathbf {L}$ is the graph Laplacian obtained in the graph construction phase. The optimization problem in (11) is essentially one natural extension of the LapRLS for multi-label cases as indicated in [35].

The minimization problem in Eq. (11) is guaranteed to have a unique global solution. The theorem for the solution in (11) are given and proved as follows.

#### Theorem 1

The minimizer of optimization problem in Eq. (11) admits an expansion

13 $$\begin{aligned} f_j^*(\mathbf {x}) = \sum _{i=1}^{n} \Theta _{ij}K(\mathbf {x}_i,\mathbf {x}), j=1,2,\ldots , L \end{aligned}$$

in terms of the labeled and unlabeled instances; $K(\cdot ,\cdot )$ represents the kernel function, which must be positive semi-definite.

#### Proof

In the multi-label classification problem (11), the norm of the function *f* can be represented by the sum of each function $f_j$ in the Reproducing Kernel Hilbert Space $\mathcal {H}_K$, i.e., $||f||_K^2 = \sum _{j=1}^{L}||f_j||_K^2$.

Any function in the RKHS $\mathcal {H}_K$ can be decomposed into two orthogonal components; specifically, each $f_j$, can be decomposed to a function $f_j^0$ in the linear subspace spanned by $\{ K(x_i,\cdot )\}_{i=1}^{n}$ and $f_j^1$ orthogonal to $f_j^0$ [3]. Accordingly, $f_j$ can be represented by

$$\begin{aligned} f_j = f_j^0 + f_j^1 = \sum _{i=1}^{n} \Theta _{ij}K(x_i,\cdot ) + f_j^1, \end{aligned}$$

Since $||f_j||_K^2=||f_j^0||_K^2+||f_j^1||_K^2\ge ||f_j^0||_K^2$, there is

$$\begin{aligned} ||f||_K^2&= \sum _{j=1}^{L}||f_j||_K^2 = \sum _{j=1}^{L}||f_j^0||_K^2\\&\quad + \sum _{j=1}^{L}||f_j^1||_K^2\ge \sum _{j=1}^{L}||f_j^0||_K^2 \end{aligned}$$

The equality is achieved if and only if $||f_j^1||_K^2=0$, $j=1,2,\ldots , L$. Therefore the minimizer must be $f_j^*(\mathbf {x}) = \sum _{i=1}^{n} \Theta _{ij}K(\mathbf {x}_i,\mathbf {x})$, $j=1,2,\ldots , L$. $\square$

Denote the $\mathbf {K}$ as a $n\times n$ matrix of the kernel estimation with respect to all the data samples $\mathbf {X}$, and $\varvec{\Theta }$ as a $n\times L$ matrix of the coefficients. The solution can be represented by

14 $$\begin{aligned} \mathbf {F}^* = \mathbf {K}\varvec{\Theta }. \end{aligned}$$

Therefore, the problem in Eq. (11) is reduced to optimizing over the finite dimensional space of coefficients $\varvec{\Theta }$. According to [3], the kernel function $K(\cdot ,\cdot )$ must be positive semi-definite which gives rise to an RKHS. A choice of the kernel function is the heat kernel, which can be approximated using a sharp Gaussian kernel. Thus, $\mathbf {U}$ in Eq. (5) can be taken as the kernel matrix $\mathbf {K}$.

### Reliance weighted kernel for performance improvement

In the framework of manifold regularization, the classifier is trained using both the labeled training set $\mathbb {D}_l$ and the unlabeled training set $\mathbb {D}_u$. Although both $\mathbb {D}_l$ and $\mathbb {D}_u$ contribute to the classification, the prediction of the label vector ${{\tilde{\mathbf{y}}}}$ of an unforeseen future sample ${{\tilde{\mathbf{x}}}}$ is based on the label information provided by the labeled training set $\mathbb {D}_l$. Naturally, this motivates us to have more trust in the labeled training set than the unlabeled one for out-of-sample prediction. Thus, a reliance weighting strategy is proposed to assign different weights to the training instances allowing samples from $\mathbb {D}_l$ to have greater influence than those from $\mathbb {D}_u$. Given a heat kernel function $K(\mathbf {x}_i,\mathbf {x})$, the weighted kernel function for $\mathbf {x}$ is

15 $$\begin{aligned} {\tilde{K}}(\mathbf {x}_i,\mathbf {x}) = K(\mathbf {x}_i,\mathbf {x}) \cdot \varXi _{i}, \end{aligned}$$

where $\varXi _{i}$ represents the reliance weight of the *i*th instance. Denote the ${{\tilde{\mathbf{K}}}}$ as the matrix of the weighted kernel estimation with respect to all the data samples $\mathbf {X}$, and the reliance weight matrix $\varvec{\varXi }$ as

16 $$\begin{aligned} \varvec{\varXi } = \left[ \begin{array}{cccc} \varXi _{1} &{}\quad 0 &{}\quad \cdots &{}\quad 0 \\ 0 &{}\quad \varXi _{2} &{}\quad \cdots &{}\quad 0 \\ \vdots &{}\quad \vdots &{}\quad \ddots &{}\quad \vdots \\ 0 &{}\quad 0 &{}\quad \cdots &{}\quad \varXi _{n}\\ \end{array} \right] \end{aligned}$$

Then, the weighted kernel matrix is ${{\tilde{\mathbf{K}}}}=\mathbf {K}\varvec{\varXi }$. To yield to the minimizer in (13), the kernel function ${\tilde{K}}(\cdot ,\cdot )$ must be positive semi-definite.

#### Proposition 1

Given a heat kernel function $K(\cdot ,\cdot )$, the weighted kernel ${\tilde{K}}(\cdot ,\cdot )=K(\cdot ,\cdot ) \cdot \varXi _{i}$ is positive semi-definite if and only if $\varXi _{i}\ge 0$.

#### Proof

Given an arbitrary vector $\mathbf {v}\in \mathbb {R}^d$, we have

17 $$\begin{aligned} \mathbf {v}^T{{\tilde{\mathbf{K}}}}\mathbf {v} = \sum _{i=1}^{d}\sum _{j=1}^{d} K(\mathbf {x}_i,\mathbf {x}_j) \cdot \varXi _{i} \cdot v_i v_j. \end{aligned}$$

where $v_i$ and $v_j$ are the *i*th and *j*th elements of $\mathbf {v}$. The kernel estimation based on a heat kernel function is always nonnegative, namely $K(\mathbf {x}_i,\mathbf {x}_j)\ge 0$. Therefore, $K(\mathbf {x}_i,\mathbf {x}_j) \cdot \varXi _{i}\ge 0$ if and only if $\varXi _{i}\ge 0$. Accordingly, $\mathbf {v}^T{{\tilde{\mathbf{K}}}}\mathbf {v} \ge 0$ if and only if $\varXi _{i}\ge 0$. As a conclusion, the weighted kernel ${\tilde{K}}(\cdot ,\cdot )=K(\cdot ,\cdot ) \cdot \varXi _{i}$ is positive semi-definite if and only if $\varXi _{i}\ge 0$. $\square$

Using the reliance weighted kernel function instead of the heat kernel function, the solution in (14) becomes

18 $$\begin{aligned} \mathbf {F}^*= {{\tilde{\mathbf{K}}}}\varvec{\Theta } = \mathbf {K}\varvec{\varXi }\varvec{\Theta }. \end{aligned}$$

The coefficient matrix $\varvec{\Theta }^*$ can be estimated by differentiating the right hand side of (11) as

$$\begin{aligned}&\frac{2}{l}\varvec{\Psi } \mathbf {K}\varvec{\varXi }(\varvec{\Psi } \mathbf {K}\varvec{\varXi } \varvec{\Theta }^*-\mathbf {Y}) + 2\gamma _A \mathbf {K}\varvec{\varXi } \varvec{\Theta }^*\\&\qquad + \frac{2\gamma _I}{n^2} (\mathbf {K}\varvec{\varXi })^T \mathbf {L} \mathbf {K}\varvec{\varXi } \varvec{\Theta }^* = 0 \end{aligned}$$

The coefficient matrix is eventually obtained as

19 $$\begin{aligned} \varvec{\Theta }^* = \left( \varvec{\Psi }\mathbf {K}\varvec{\varXi } + l\gamma _A\mathbf {I} + \frac{l\gamma _I}{n^2} \mathbf {L} \mathbf {K} \varvec{\varXi } \right) ^{-1} \mathbf {Y}. \end{aligned}$$

where $\mathbf {I}$ is a $n\times n$ identity matrix.

For unforeseen future samples ${{\tilde{\mathbf{X}}}}=[{{\tilde{\mathbf{x}}}}_1,{{\tilde{\mathbf{x}}}}_2,\ldots , {{\tilde{\mathbf{x}}}}_e]^T$ in $\mathbb {D}_e$, the label matrix ${{\tilde{\mathbf{F}}}}$ is obtained as follows: first, a $e\times n$ kernel matrix $\mathbf {K}_e$ is calculated using Eq. (5), i.e., ${\tilde{K}}_{ij} = K({{\tilde{\mathbf{x}}}}_i, \mathbf {x}_j)$ for $i=1,2,\ldots ,e$ and $j=1,2,\ldots ,n$. Next, the output ${{\tilde{\mathbf{F}}}}$ for ${{\tilde{\mathbf{X}}}}$ can be calculated as

20 $$\begin{aligned} {{\tilde{\mathbf{F}}}} = \mathbf {K}_e\varvec{\varXi }\varvec{\Theta }^*. \end{aligned}$$

Eventually, the label matrix ${{\tilde{\mathbf{Y}}}}$ of ${{\tilde{\mathbf{X}}}}$ is obtained by comparing each element of ${{\tilde{\mathbf{F}}}}$ with 0. We will henceforth refer to our multi-label extension of MR as Multi-Label Manifold Regularization (ML-MR), and our reliance weighting augmentation as ML-MR with Reliance Weighting (ML-MRRW).

There are clearly many strategies for determining reliance weights. The simplest strategy is to assign uniform weights, namely $\varXi _{i}=\nu _1\in [0,1], 1\le i \le l$ and $\varXi _{i}=\nu _2\in [0,1], l< i \le l+u$ for all labeled and unlabeled training instances, respectively. These two parameters then decide the balance of trust between labeled and unlabeled training data. The extended manifold regularization is supervised if $\nu _1=1$ and $\nu _2=0$ are used, and is unsupervised for the choice of $\nu _1=0$ and $\nu _2=1$. The relation $\nu _1=\nu _2$ indicates that the impacts of $\mathbb {D}_l$ and $\mathbb {D}_u$ to label inference are equal, whereas $\nu _1>\nu _2$ indicates that more weight is put on labeled instances $\mathbb {D}_l$ than that on unlabeled instances $\mathbb {D}_u$. In this work, we are trying to improve the performance of manifold regularization by trusting labeled instances more, and thus the choices of $\nu _1$ and $\nu _2$ must follow two criterions, namely $\nu _1=1$ and $\nu _1>\nu _2>0$.

## Experimental design

This section designs experiments to validate the effectiveness of the proposed ML-MR and ML-MRRW methods on some commonly used benchmark data sets. Other semi-supervised multi-label classification methods are tested as comparisons, across a range of performance metrics.

### Data sets

Four public data sets from different domains are chosen for the experimental study. Table 1 presents the basic information about these data sets. The first data set “Emotions” [52] consists of sampled wave forms of sound clips generated from different genres of musical songs. Each instance is labeled with 6 emotions: amazed-surprised, happy-pleased, relaxing-calm, quiet-still, sad-lonely, and angry-aggressive. The second data set “Scene” [7] is a commonly used image data set with each image represented by a 294-dimension feature vector and labeled with six classes: beach, sunset, field, fall-foliage, mountain, and urban. The third data set “Yeast” [19] consists of micro-array expression data and phylogenetic profiles for 2107 genes. Each gene is associated with a set of functional classes, which are grouped into 14 functional categories. The last data set “mediamill” [46] consists of digital video achieves for the TREC Video Retrieval Evaluation (TRECVID) challenge. This data set contains 120 features and 101 annotation concepts. These data sets are already formatted, so no further pre-processing is needed.

**Table 1:** Basic information of the selected public data sets

Data set	Domain	# Features	# Labels	# Instances
Emotions [52]	Music	72	6	593
Scene [7]	Image	294	6	2409
Yeast [19]	Life	103	14	2417
Mediamill [46]	Video	120	101	43,907

### Experiment setup

In each experiment, the data set is first partitioned into two parts: the training data and out-of-sample testing data occupy two thirds and one third of the whole data set, respectively. Then, the labels of a portion of the instances in the training data are omitted to construct labeled training data and unlabeled training data. The labeling rate $\eta$ is drawn from {5%, 10%, 15%, 20%, 25%, 30%, 35%, 40%, 45%, 50%}. For each labeling rate, experiments are conducted 100 times by randomly resampling the labeled training data, unlabeled training data, and out-of-sample testing data. The first three data sets “Emotions”, “Scene”, and “Yeast” are fully used in the experiments, whereas only a portion (10% randomly selected) of the “Mediamill” data is used in view of the computational complexity of MR.

In the experiments, seven algorithms are carried out for comparisons: (1) the Multi-Label k Nearest Neighbors (MLkNN) [57], (2) the Multi-Label Gaussian Fields and Harmonic Functions (ML-GFHF) [56], (3) the Multi-Label Local and Global Consistency (ML-LGC) [56], (4) the Fixed-Size Multi-Label Regularized Kernel Spectral Clustering (ML-FSKSC) [33], (5) the Semi-Supervised Weak-Label approach (SSWL) [18], (6) the Multi-Label Manifold Regularization (ML-MR), and (7) the ML-MR with the Reliance Weighting strategy (ML-MRRW) in “Reliance weighted kernel for performance improvement”. It should be noted that all the seven algorithms are applied in the first three experiments. In the last experiment, only six algorithms are applied; the SSWL is not included in the comparison because the used personal computer failed to run the algorithm owing to the high computational burden. Among all of the algorithms, MLkNN is supervised and all the other algorithms are semi-supervised. Accordingly, the MLkNN algorithm only uses the labeled training data in the training phase, whereas all the other algorithms exploit both the labeled training data and unlabeled training data. The parameters in each algorithm are determined by parameter exploration using a small portion of the data. For the ML-MRRW algorithm, the two parameters for the reliance weighting strategy are fixed at $[\nu _1,\nu _2]=[1,0.1]$.

### Performance metrics

Many performance metrics or criteria for multi-label classification have been proposed; reviews may be found in [47] and [58]. In this work, three popular metrics are used to evaluate the performances of the algorithms in learning multi-label problems.

The average precision calculates the average fraction of labels ranked above a particular label that are truly predicted. The larger the value of it, the better the learning performance:

21 $$\begin{aligned}&\text{ avgprec }(f)= \frac{1}{n} \sum _{i=1}^n \frac{1}{|\mathbf {y_i}|} \sum _{y_{ij}\in \mathbf {y_i}} \nonumber \\&\qquad \frac{|\{ y'_{ij}|\text{ rank}_f(\mathbf {x}_i,y'_{ij})\le \text{ rank}_f(\mathbf {x}_i,y_{ij}),y'_{ij}\in \mathbf {y}_i \}|}{\text{ rank}_f(\mathbf {x}_i,y_{ij})} \end{aligned}$$

where $y'_i$ is the chosen particular label. $y_{ij}$ is the *j*th label of instance *i*.

*F*1 is a popular measure for single label. It is the harmonic mean of precision and recall:

22 $$\begin{aligned} F1 = \frac{2\times tp}{2\times tp+fp+fn} \end{aligned}$$

where *tp* is the number of true positives, *tn* is the number of true negatives, *fp* is the number of false positives, and *fn* is the number of false negatives. Macro-*F*1 and Micro-*F*1 are multi-label classifier metrics derived by computing the *F*1 measure across the label set; either after summing true and false positives and false negatives across all labels, or by averaging the *F*1 measure for each label:

23 $$\begin{aligned}&F1_{micro}=F1\left( \sum _{\lambda =1}^{L}tp_\lambda ,\sum _{\lambda =1}^{L}fp_\lambda , \sum _{\lambda =1}^{L}fn_\lambda \right) \end{aligned}$$

24 $$\begin{aligned}&F1_{macro}=\frac{1}{L}\sum _{\lambda =1}^L F1 \left( tp_\lambda ,fp_\lambda ,fn_\lambda \right) \end{aligned}$$

where $tp_\lambda$ is the number of true positives, $fp_\lambda$ is the number of false positives, and $fn_\lambda$ is the number of false negatives of label $\lambda$ after being evaluated by binary evaluation of *F*1. Larger values of $F1_{micro}$ and $F1_{macro}$ denote better performance.

### Significance test

Statistical tests are commonly used to ensure that differences between machine-learning algorithms are meaningful [15, 23, 44]. In this paper, the Friedman test and a post hoc test are utilized. Friedman’s Test is a simple and robust nonparametric method for testing the differences between multiple algorithms over multiple data sets. It ranks the algorithms from the smallest rank to the largest rank based on their performance scores for each data set separately, and average ranks are assigned to ties. For instance, the best performing algorithm is assigned rank 1, the second best performing algorithm is assigned rank 2, $\ldots$. Denote $R_{i}$ as the sum of ranks for the *i*th algorithm ($i=1,2,3, \ldots , K$) over *N* different data sets. Then, the Friedman’s statistic $F_{R}$ [22, 44] is given by

25 $$\begin{aligned} F_{R}=\frac{12}{NK(K+1)}\sum _{i=1}^{K} R_{i}^2 -3N(K+1). \end{aligned}$$

The null hypothesis $H_{0}$ is that there are no significant differences between the algorithms, the alternative hypothesis $H_{1}$ is that there are significant differences between the algorithms. $F_{R}$ tests the null hypothesis $H_{0}$ against the alternative hypothesis $H_{1}$. For *K* larger than 5, the distribution of $F_{R}$ can be approximated by a Chi-square distribution with $K-1$ degree of freedom. Thus, for any pre-chosen $\alpha$ level of significance, the null hypothesis $H_{0}$ is rejected if $F_{R}>\chi _{\alpha }^2$. In this paper, there are 7 algorithms applied to the first three data sets, so $K-1=6$. Thus, the critical Chi-square value is $\chi _{\alpha }^2=12.592$ given $\alpha =0.05$. There are six algorithms carried out to the last data set, namely Mediamill, so $K-1=5$. Thus, the critical Chi-square value is $\chi _{\alpha }^2=11.070$ given $\alpha =0.05$.

When the null hypothesis is rejected, the analysis continues with a post hoc test [44]. Denote the difference $D_{ij}=R_{i}-R_{j}$ between the rank sums of algorithms *i* and *j*. The performance of two algorithms is significantly different if the difference $|D_{ij}|$ between their corresponding rank sums is no less than the *critical difference*

26 $$\begin{aligned} CD=z\sqrt{ \frac{NK(K+1)}{6} }, \end{aligned}$$

where *z* is the z-score from the standard normal curve corresponding to $\frac{\alpha }{K(K-1)}$, and $\alpha$ is the level of significance. It can be concluded that the performance of the algorithm *i* is significantly better than that of the algorithm *j*, if $|D_{ij}|\ge CD$ and $D_{ij}<0$; otherwise, worse, if $|D_{ij}|\ge CD$ and $D_{ij}>0$.

**Fig. 1:**
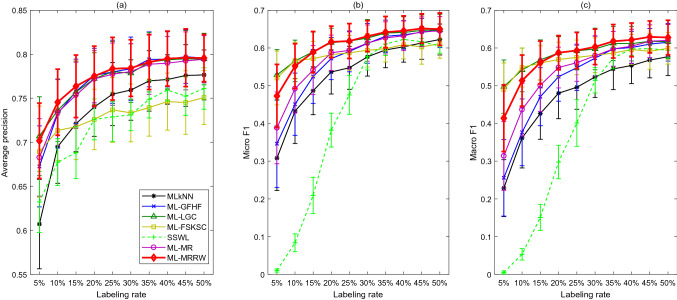
Performance metrics vs. labeling rates for seven classification algorithms applied to the “Emotions” data

## Experimental results and discussion

We compare the proposed ML-MR and ML-MRRW against four well-known semi-supervised, and one supervised, multi-label algorithms on the chosen data sets. When calculating the Friedman’s statistic test and post hoc statistic test for each data set, the ten sampled data sets under each labeling rate (from 5 to $50\%$) are considered as different data sets.

### Case I: Emotions

The experimental results for the “Emotions” data are shown in Fig. 1. The sub-figures from left to right present the A-precision (A-precision stands for average precision), Micro-F1, and Macro-F1 for all the algorithms under different labeling rates, respectively. The error bars indicate one standard deviation of the metrics. Table 2 presents the calculated Friedman’s statistics $F_{R}$ based on ranking scores for the three different performance metrics; all of them are greater than the critical Chi-square value $\chi _{\alpha }^2=12.592$. Thus, the null hypothesis is rejected, and it can be concluded that there are significant differences between the performances of the seven algorithms.

**Table 2:** The Friedman’s statistics $F_{R}$ for different performance metrics in Case I

	A-precision	Micro-F1	Macro-F1
$F_R$	49.9714	46.2857	47.9143

**Table 3:** The differences between the rank sums of the ML-MRRW and the other algorithms in Case I (MLkNN, ML-GFHF, ML-LGC, ML-FSKSC, SSWL, ML-MR, and ML-MRRW are denoted by algorithms 1, 2, 3, 4, 5, 6, and 7)

	A-precision	Micro-F1	Macro-F1
$D_{71}$	– 39	– 46	– 49
$D_{72}$	– 9	– 25	– 30
$D_{73}$	– 13	– 1	– 5
$D_{74}$	– 46	– 29	– 22
$D_{75}$	– 51	– 47	– 49
$D_{76}$	– 24	– 20	– 20

**Table 4:** Comparison with the state-of-the-art literature [31] on the “Emotions” data

	BR	CC	CLR	QWML	HOMER	ML-C4.5	PCT	ML-KNN	RAKEL	ECC	RFML-C4.5	RF-PCT	ML-MRRW (50%)	ML-MRRW (70%)
A-precision	0.721	0.724	0.718	0.679	0.698	0.759	0.713	0.649	0.713	0.687	0.812	0.812	0.796	0.855
Micro-F1	0.509	0.503	0.512	0.528	0.588	0.655	0.571	0.457	0.533	0.554	0.647	0.672	0.650	0.727
Macro-F1	0.440	0.420	0.443	0.458	0.570	0.630	0.568	0.385	0.488	0.500	0.620	0.650	0.628	0.695

The values in the brackets denote the labeling rates of the data used by ML-MRRW

**Table 5:** Comparison with supervised multi-label ensemble algorithms in [37] on the “Emotions” data

	EBR	ECC	$MLS_{\text {train}}$	HOMER	AdaB.MH	ELP	EPS	RAkEL2	TREMLC	CDE	RF-PCT	CBMLC	ML-MRRW (50%)	ML-MRRW (70%)
Micro-F1	0.653	0.666	0.599	0.572	0.105	0.660	0.654	0.648	0.628	0.652	0.671	0.557	0.650	0.727
Macro-F1	0.633	0.650	0.592	0.564	0.059	0.642	0.637	0.633	0.616	0.637	0.653	0.547	0.628	0.695

Further, post hoc test is carried out. The differences between the rank sums of the ML-MRRW and the other algorithms are calculated and presented in Table 3. Denote MLkNN, ML-GFHF, ML-LGC, ML-FSKSC, SSWL, ML-MR, and ML-MRRW by algorithms 1, 2, 3, 4, 5, 6, and 7, respectively. Then, $D_{7i},i=1,2,\ldots ,6$ represents the difference between rank sums of the ML-MRRW and the *i*th algorithm. The critical difference for $K=7$ and $\alpha = 0.05$ is $CD=9.2815$. For each performance metric, any difference value $|D_{7i}|\ge CD$ indicates a significant difference between ML-MRRW and the algorithm *i* with respect to this metric. Further, $|D_{7i}|\ge CD$ and $D_{7i}<0$ indicate ML-MRRW outperforms the algorithm *i*. From Table 3, $D_{71}$, $D_{73}$, $D_{74}$, $D_{75}$ and $D_{76}$ are less than 0 and their absolute values are larger than the critical value $CD=9.2815$ with respect to A-precision; thus, ML-MRRW outperforms MLkNN, ML-LGC, ML-FSKSC, SSWL, and ML-MR in terms of A-precision. Moreover, $D_{71}$, $D_{72}$, $D_{74}$, $D_{75}$ and $D_{76}$ are less than 0 and their absolute values are larger than the critical value $CD=9.2815$ with respect to Micro-F1 and Macro-F1; thus, it outperforms MLkNN, ML-GFHF, ML-FSKSC, SSWL, and ML-MR in terms of Micro-F1 and Macro-F1.

In general, the following conclusions can be drawn from the plots and tables:

1. SSWL does not work well under low labeling rates, however, it improves the performance very much as labeling rate increases. It works almost the same as MLkNN as labeling rate higher than $30\%$. The other five semi-supervised multi-label learning algorithms show much better overall performances compared to the MLkNN and SSWL methods, except that ML-FSKSC has lower A-precision for large labeling rates.
2. The ML-MRRW algorithm has the highest A-precision, Micro-F1, and Macro-F1 among all the multi-label learning algorithms for most of the labeling rates. Specifically, it defeats all the other approaches except ML-GFHF in terms of A-precision, and it outperforms all the other methods except ML-LGC regarding Micro-F1 and Macro-F1.
3. Overall, ML-MRRW outperforms all the other algorithms.

Moreover, ML-MRRW is also compared with supervised multi-label algorithms from the state-of-the-art literature [31], and supervised multi-label ensemble algorithms in [37] on the “Emotions” data in Tables 4 and 5, respectively. The performance metrics include the mean values of A-precision, Micro-F1, and Macro-F1. The second last column presents the three metrics achieved by ML-MRRW under the labeling rate of $50\%$ (also shown in Fig. 1). It can be found that ML-MRRW under this labeling rate outperforms most algorithms in terms of A-precision, Micro-F1, and Macro-F1. It also outperforms some ensemble algorithms, including $MLS_{\text {train}}$, HOMER, AdaB.MH, TREMLC, and CBMLC, and it does almost as well as the other ensemble methods in Table 5 under the 50% labeling rate. The last column presents the metrics as the labeling rate increases to $70\%$; at this labeling rate, ML-MRRW is found to outperform all of the baselines in both Tables 4 and  5.

**Fig. 2:**
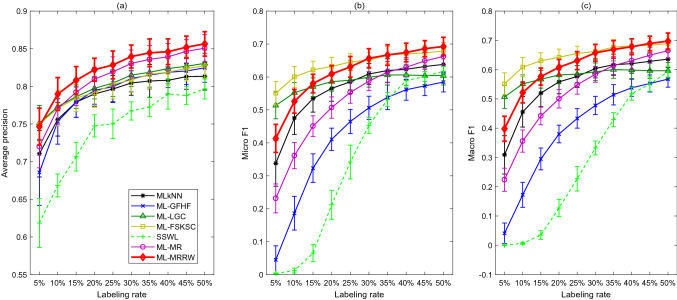
Performance metrics vs. labeling rates for seven classification algorithms applied to the “Scene” data

### Case II: Scene

The experimental results for the “Scene” data are shown in Fig. 2. Table 6 presents the calculated Friedman’s statistics $F_{R}$ according to ranking scores for the three different performance metrics. It can be found that all of them are greater than the critical Chi-square value $\chi _{\alpha }^2=12.592$. Thus, the null hypothesis is rejected, and it can be concluded that there are significant differences between the performances of the seven algorithms. Further, the differences between the rank sums of the ML-MRRW and the other algorithms are calculated and presented in Table 7. From Table 7, $D_{71}$, $D_{72}$, $D_{73}$, $D_{74}$, $D_{75}$ and $D_{76}$ are less than 0 and their absolute values are larger than the critical value $CD=9.2815$ with respect to A-precision; thus, ML-MRRW outperforms MLkNN, ML-GFHF, ML-LGC, ML-FSKSC, SSWL and ML-MR in terms of A-precision. Moreover, $D_{71}$, $D_{72}$, $D_{73}$, $D_{75}$ and $D_{76}$ are less than 0 and their absolute values are larger than the critical value $CD=9.2815$ with respect to Micro-F1 and Macro-F1; thus, it outperforms MLkNN, ML-GFHF, ML-LGC, SSWL and ML-MR in terms of Micro-F1 and Macro-F1.

Generally, the following conclusions can be drawn from the plots and tables:

1. SSWL works worse than the other approaches. It does not work well under low labeling rates, but it improves the performance a lot as labeling rate increases.
2. The A-precision of ML-LGC, ML-GFHF, ML-FSKSC, and MLkNN, are quite close, whereas the ML-MR and ML-MRRW have significantly larger values on this metric under different labeling rates.
3. ML-MRRW defeats all the other algorithms in terms of A-precision, and it outperforms all the other approaches except ML-FSKSC regarding Micro-F1 and Macro-F1.
4. Overall, ML-MRRW performs better than ML-FSKSC in terms of A-precision. ML-FSKSC and ML-MRRW achieve the best performances in terms of Micro-F1 and Macro-F1. ML-MRRW performs better than ML-FSKSC in terms of Micro-F1 and Macro-F1 under high labeling rates and worse under low labeling rates.

Moreover, ML-MRRW is also compared with supervised multi-label algorithms from the state-of-the-art literature [31] and supervised multi-label ensemble algorithms in [37] on the “Scene” data in Tables 8 and 9, respectively. The second last column presents the mean values of A-precision, Micro-F1, and Macro-F1 for ML-MRRW under the labeling rate 50% (also shown in Fig. 2). From Table 8, ML-MRRW under this labeling rate outperforms HOMER, ML-C4.5, PCT, and ML-KNN in terms of A-precision, outperforms ML-C4.5, PCT, ML-KNN, and RF-PCT in terms of Macro-F1, and outperforms ML-C4.5, PCT, RFML-C4.5 and RF-PCT in terms of Micro-F1. It also outperforms some ensemble algorithms, including $MLS_{train}$, HOMER, AdaB.MH, and CBMLC, and it does almost as well as the other ensemble methods in Table 9. The last column presents the metrics as the labeling rate increases to $90\%$; at this level, ML-MRRW is found to outperform all the baselines in both Tables 8 and  9.

**Table 6:** The Friedman’s statistics $F_{R}$ for different performance metrics in Case II

	A-precision	Micro-F1	Macro-F1
$F_R$	54	50.9143	53.3143

**Table 7:** The differences between the rank sums of the ML-MRRW and the other algorithms in Case II (MLkNN, ML-GFHF, ML-LGC, ML-FSKSC, SSWL, ML-MR, and ML-MRRW are denoted by algorithms 1, 2, 3, 4, 5, 6, and 7)

	A-precision	Micro-F1	Macro-F1
$D_{71}$	– 45	– 21	– 18
$D_{72}$	– 39	– 46	– 41
$D_{73}$	– 15	– 21	– 17
$D_{74}$	– 27	2	8
$D_{75}$	– 58	– 49	– 49
$D_{76}$	– 12	– 26	– 23

**Fig. 3:**
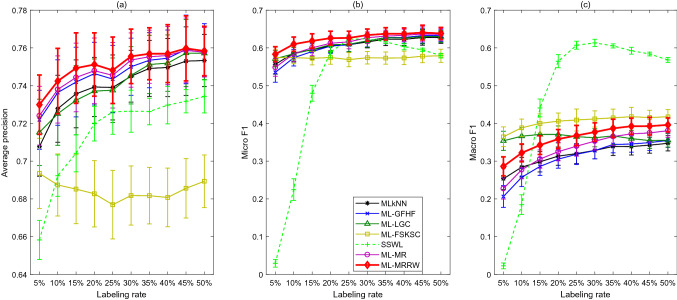
Performance metrics vs. labeling rates for seven classification algorithms applied to the “Yeast” data

**Table 8:** Comparison with the state-of-the-art literature [31] on the “Scene” data

	BR	CC	CLR	QWML	HOMER	ML-C4.5	PCT	ML-KNN	RAKEL	ECC	RFML-C4.5	RF-PCT	ML-MRRW (50%)	ML-MRRW (90%)
A-precision	0.893	0.881	0.886	0.864	0.848	0.751	0.745	0.851	0.862	0.856	0.862	0.874	0.856	0.899
Micro-F1	0.761	0.757	0.758	0.756	0.764	0.593	0.516	0.661	0.772	0.762	0.717	0.669	0.697	0.775
Macro-F1	0.765	0.762	0.762	0.759	0.768	0.596	0.593	0.692	0.777	0.770	0.514	0.658	0.692	0.778

**Table 9:** Comparison with supervised multi-label ensemble algorithms in [37] on “Scene” data

	EBR	ECC	$MLS_{train}$	HOMER	AdaB.MH	ELP	EPS	RAkEL2	TREMLC	CDE	RF-PCT	CBMLC	ML-MRRW (50%)	ML-MRRW (90%)
Micro-F1	0.702	0.722	0.638	0.576	0.000	0.697	0.696	0.693	0.692	0.714	0.702	0.591	0.697	0.775
Macro-F1	0.706	0.729	0.647	0.586	0.000	0.704	0.703	0.701	0.700	0.720	0.711	0.598	0.692	0.778

### Case III: Yeast

The experimental results for the “Yeast” data are shown in Fig. 3. Table 10 presents the calculated Friedman’s statistics $F_{R}$ for the three different performance metrics. It can be found that all of them are greater than the critical Chi-square value $\chi _{\alpha }^2=12.592$. Thus, the null hypothesis is rejected, and it can be concluded that there are significant differences between the performances of the 7 algorithms. Further, the differences between the rank sums of the ML-MRRW and the other algorithms are calculated and presented in Table 11. From Table 11, $D_{71}$, $D_{72}$, $D_{73}$, $D_{74}$, $D_{75}$ and $D_{76}$ are less than 0 and their absolute values are larger than the critical value $CD=9.2815$ with respect to A-precision and Micro-F1; thus, ML-MRRW outperforms MLkNN, ML-GFHF, ML-LGC, ML-FSKSC, SSWL and ML-MR in terms of A-precision and Micro-F1. Moreover, $D_{71}$, $D_{72}$ and $D_{76}$ are less than 0 and their absolute values are larger than the critical value $CD=9.2815$ with respect to Macro-F1; thus, it outperforms MLkNN, ML-GFHF and ML-MR in terms of Macro-F1.

**Table 10:** The Friedman’s statistics $F_{R}$ for different performance metrics in Case III

	A-precision	Micro-F1	Macro-F1
$F_R$	57.1714	41.2286	40.8429

**Table 11:** The differences between the rank sums of the ML-MRRW and the other algorithms in Case III (MLkNN, ML-GFHF, ML-LGC, ML-FSKSC, SSWL, ML-MR, and ML-MRRW are denoted by algorithms 1, 2, 3, 4, 5, 6, and 7)

	A-precision	Micro-F1	Macro-F1
$D_{71}$	– 35	– 35	– 28
$D_{72}$	– 16	– 35	– 32
$D_{73}$	– 33	– 27	– 5
$D_{74}$	– 58	– 52	14
$D_{75}$	– 50	– 46	10
$D_{76}$	– 11	– 15	– 15

**Table 12:** Comparison with the state-of-the-art literature [31] on the “Yeast” data

	BR	CC	CLR	QWML	HOMER	ML-C4.5	PCT	ML-KNN	RAKEL	ECC	RFML-C4.5	RF-PCT	ML-MRRW (50%)	ML-MRRW (75%)
A-precision	0.722	0.727	0.719	0.718	0.663	0.620	0.705	0.732	0.715	0.667	0.738	0.744	0.758	0.786
Micro-F1	0.652	0.650	0.655	0.654	0.673	0.610	0.577	0.625	0.656	0.658	0.593	0.617	0.638	0.675
Macro-F1	0.392	0.390	0.392	0.394	0.447	0.370	0.293	0.336	0.359	0.350	0.283	0.322	0.396	0.462

**Table 13:** Comparison with supervised multi-label ensemble algorithms in [37] on “Yeast” data

	EBR	ECC	$MLS_{train}$	HOMER	AdaB.MH	ELP	EPS	RAkEL2	TREMLC	CDE	RF-PCT	CBMLC	ML-MRRW (50%)	ML-MRRW (75%)
Micro-F1	0.626	0.637	0.548	0.585	0.480	0.626	0.625	0.621	0.609	0.631	0.636	0.493	0.638	0.675
Macro-F1	0.387	0.401	0.395	0.403	0.122	0.380	0.375	0.409	0.389	0.410	0.396	0.396	0.396	hl0.462

In general, the following conclusions can be drawn from the plots and tables:

1. SSWL does not work well under low labeling rates, but it improves the performance a lot as labeling rate increases. Furthermore, it outperforms the other methods with labeling rate higher than $15\%$ in terms of Macro-F1.
2. The ML-MRRW and ML-MR algorithms have the best performances in terms of the A-precision and Micro-F1 for all the labeling rates.
3. ML-MRRW has the superior performance among all the algorithms in terms of Micro-F1 and A-precision, but it performs worse than ML-FSKSC under all labeling rates considering Macro-F1. It performs worse than SSWL and ML-LGC with high labeling rates and low labeling rates, respectively.

Moreover, ML-MRRW is also compared with supervised multi-label algorithms from the state-of-the-art literature [31] and supervised multi-label ensemble algorithms in [37] on the “Yeast” data in Tables 12 and 13, respectively. The second last column presents the mean values of the A-precision, Micro-F1, and Macro-F1 for ML-MRRW under the labeling rate 50% (also shown in Fig. 3). From Table 12, ML-MRRW under this labeling rate outperforms all the algorithms in terms of A-precision, outperforms ML-C4.5, PCT, ML-KNN, RFML-C4.5, and RF-PCT in terms of Micro-F1, and it outperforms all the algorithms except for HOMER in terms of Micro-F1. It also outperforms some ensemble algorithms, including EBR, $MLS_{\text {train}}$, AdaB.MH, ELP, EPS, TREMLC, RF-PCT, and CBMLC, and it does almost as well as the other ensemble methods in Table 13. The last column presents the metrics as the labeling rate increases to $75\%$; at this level, ML-MRRW is found to outperform all the baselines in both Tables 12 and 13.

**Fig. 4:**
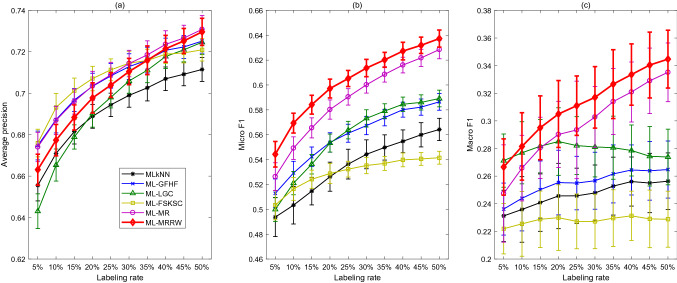
Performance metrics vs. labeling rates for six classification algorithms applied to the “Mediamill” data

### Case IV: Mediamill

The experimental results for the “Mediamill” data are shown in Fig. 4. Table 14 presents the calculated Friedman’s statistics $F_{R}$ for the three different performance metrics. It can be found that all of them are greater than the critical Chi-square value $\chi _{\alpha }^2=11.070$. Thus, the null hypothesis is rejected, and it can be concluded that there are significant differences between the performances of the six algorithms.

Further, the differences between the rank sums of the ML-MRRW and the other algorithms are calculated and presented in Table 15. Denote MLkNN, ML-GFHF, ML-LGC, ML-FSKSC, ML-MR, and ML-MRRW by algorithms 1, 2, 3, 4, 5, and 6, respectively. Then, $D_{6i},i=1,2,\ldots ,5$ represents the difference between rank sums of the ML-NRRW and the *i*th algorithm. The critical difference for $K=6$ and $\alpha = 0.05$ is $CD=7.7658$. For each performance metric, any difference value $|D_{6i}|\ge CD$ indicates a significant difference between ML-MRRW and the algorithm *i* with respect to this metric. Further, $|D_{6i}|\ge CD$ and $D_{6i}<0$ indicate ML-MRRW outperforms the algorithm *i*. From Table 15, $D_{61}$ and $D_{63}$ are less than 0 and their absolute values are larger than the critical value $CD=9.2815$ with respect to A-precision; thus, ML-MRRW outperforms MLkNN and ML-LGC in terms of A-precision. Moreover, $D_{61}$, $D_{62}$, $D_{63}$, $D_{64}$ and $D_{65}$ are less than 0 and their absolute values are larger than the critical value $CD=9.2815$ with respect to Micro-F1 and Macro-F1; thus, it outperforms all the other algorithms in terms of Micro-F1 and Macro-F1.

Generally, the following conclusions can be drawn from the plots and tables:

1. From the sub-figure of A-precision, the ML-MRRW and ML-MR outperform MLkNN and ML-LGC. They perform better than ML-GFHF and ML-FSKSC with high labeling rates but worse than them with low labeling rates.
2. From the sub-figures of Micro-F1 and Macro-F1, it can be seen that the ML-MR and ML-MRRW methods outperform all the other methods quite a lot under all labeling rates. Especially, the ML-MRRW method achieves the best performances regarding these two metrics.
3. Overall, ML-MRRW shows superior performances over all the other algorithms with Micro-F1 and Macro-F1 and it illustrates great potential for high-dimensional data sets with large number of labels.

Moreover, ML-MRRW is also compared with supervised multi-label algorithms from the state-of-the-art literature [31] and supervised multi-label ensemble algorithms in [37] on the “Mediamill” data in Tables 16 and 17, respectively. Note that these experiments in the literature consider the whole Mediamill data set, as opposed to a randomly selected subset (redrawn for each experimental run) as in our work. The second last column presents the mean values of the A-precision, Micro-F1, and Macro-F1 for ML-MRRW under the labeling rate 50% (also shown in Fig. 4). From Table 16, ML-MRRW under this labeling rate outperforms all algorithms in terms of the three metrics, except for RF-PCT in terms of A-precision. It is also superior to all the supervised ensemble algorithms in [37] from Table 17. The last column presents the metrics as the labeling rate increases to $65\%$; at this level, ML-MRRW is found to outperform all the baselines in both Tables 16 and 17.

**Table 14:** The Friedman’s statistics $F_{R}$ for different performance metrics in Case IV

	A-precision	Micro-F1	Macro-F1
$F_R$	34.3429	46.0571	47.8857

**Table 15:** The differences between the rank sums of the ML-MRRW and the other algorithms in Case IV (MLkNN, ML-GFHF, ML-LGC, ML-FSKSC, ML-MR, and ML-MRRW are denoted by algorithms 1, 2, 3, 4, 5 and 6)

	A-precision	Micro-F1	Macro-F1
$D_{61}$	– 24	– 44	– 39
$D_{62}$	7	– 26	– 29
$D_{63}$	– 18	− 25	– 15
$D_{64}$	9	– 45	– 49
$D_{65}$	14	– 10	– 12

**Table 16:** Comparison with the state-of-the-art literature [31] on the “Mediamill” data

	BR	CC	CLR	QWML	HOMER	ML-C4.5	PCT	ML-KNN	RAKEL	ECC	RFML-C4.5	RF-PCT	ML-MRRW (50%)	ML-MRRW (65%)
A-precision	0.686	0.672	0.450	0.492	0.583	0.669	0.654	0.703	0.492	0.453	0.728	0.737	0.730	0.771
Micro-F1	0.533	0.509	0.118	0.119	0.553	0.007	0.477	0.545	0.440	0.453	0.546	0.563	0.637	0.655
Macro-F1	0.056	0.052	0.037	0.037	0.073	0.003	0.031	0.113	0.019	0.022	0.088	0.112	0.345	0.432

**Table 17:** Comparison with supervised multi-label ensemble algorithms in [37] on “Mediamill” data

	EBR	ECC	$MLS_{train}$	HOMER	AdaB.MH	ELP	EPS	RAkEL2	TREMLC	CDE	RF-PCT	CBMLC	ML-MRRW (50%)	ML-MRRW (65%)
Micro-F1	0.617	0.616	0.555	0.549	0.287	DNF	0.600	0.618	0.300	DNF	0.621	0.110	0.637	0.655
Macro-F1	0.187	0.179	0.211	0.175	0.009	DNF	0.164	0.233	0.033	DNF	0.200	0.074	0.345	0.432

## Conclusion

This paper studies the semi-supervised multi-label classification problem, and extends the graph-based manifold regularization to the multi-label case. The proposed method includes three essential components, including the graph construction, the manifold regularization with multiple labels, and the exploitation of a reliance weighting strategy. This last component is intended to improve the learning ability by assigning higher weights to labeled training set and lower weights to unlabeled training sets. Extensive experiments are conducted on four public data sets with different categories to test the performances of the proposed Multi-Label Manifold Regularization (ML-MR), both with and without the Reliance Weighting (RW) strategy. Other well-known semi-supervised and supervised multi-label algorithms are tested as comparisons. Generally, the experimental results show that the proposed ML-MRRW algorithm has overall better performance than all the other algorithms under different labeling rates. In addition, ML-MRRW shows better performance than ML-MR, indicating the proposed reliance weighting strategy is effective in improving the learning performance of the ML-MR method. Further, unlike the other algorithms, ML-MRRW works consistently well on all the data sets. Also ML-MRRW is compared with 12 supervised multi-label algorithms and 12 ensemble approaches from the literature on the public data sets. As evidenced by the results, ML-MRRW outperforms all the baselines by supervised methods on these data sets. All in all, ML-MRRW is a promising semi-supervised multi-label algorithm for classification.

## Data Availability

Not applicable.

## References

[1] Ashfaq RAR, Wang XZ, Huang JZ, Abbas H, He YL (2017). Fuzziness based semi-supervised learning approach for intrusion detection system. Inf Sci.

[2] Belkin M, Niyogi P (2004). Semi-supervised learning on Riemannian manifolds. Mach Learn.

[3] Belkin M, Niyogi P, Sindhwani V (2006). Manifold regularization: a geometric framework for learning from labeled and unlabeled examples. J Mach Learn Res.

[4] Belkin M, Niyogi P (2003) Using manifold structure for partially labeled classification. Adv Neural Inf Process Syst 953–960

[5] Blum A, Chawla S (2001) Learning from labeled and unlabeled data using graph mincuts. In: Proc. 18th International Conf. on Machine Learning, pp 19–26

[6] Blum A, Mitchell T (1998) Combining labeled and unlabeled data with co-training. In: Proceedings of the Eleventh Annual Conference on Computational Learning Theory, pp 92–100. ACM

[7] Boutell MR, Luo J, Shen X, Brown CM (2004). Learning multi-label scene classification. Pattern Recogn.

[8] Carmeli C, De Vito E, Toigo A (2006). Vector valued reproducing kernel Hilbert spaces of integrable functions and Mercer theorem. Anal Appl.

[9] Cevikalp H, Franc V (2017). Large-scale robust transductive support vector machines. Neurocomputing.

[10] Chapelle O, Scholkopf B, Zien A (2006). Semi-supervised learning.

[11] Chapelle O, Sindhwani V, Keerthi SS (2008). Optimization techniques for semi-supervised support vector machines. J Mach Learn Res.

[12] Chapelle O, Zien A (2005) Semi-supervised classification by low density separation. In: AISTATS, pp 57–64. Citeseer

[13] Collobert R, Sinz F, Weston J, Bottou L (2006). Large scale transductive svms. J Mach Learn Res.

[14] Dempster AP, Laird NM, Rubin DB (1977) Maximum likelihood from incomplete data via the EM algorithm. J Roy Stat Soc Ser B (methodological) 1–38

[15] Demšar J (2006). Statistical comparisons of classifiers over multiple data sets. J Mach Learn Res.

[16] Ding S, Zhu Z, Zhang X (2017). An overview on semi-supervised support vector machine. Neural Comput Appl.

[17] Dirichlet PGL, des Satzes B (1837) dass jede unbegrentze arithmetische progression, deren erstes glied und differenz ganze zahlen ohne gemeinschaftlichen factor sind, unendlich viele primzahlen enth alt. Abh. der Königlichen Preuss. Akad. der Wiss, pp 45–81

[18] Dong HC, Li YF, Zhou ZH (2018) Learning from semi-supervised weak-label data. In: 32nd AAAI Conference on Artificial Intelligence, AAAI 2018, pp 2926–2933. New Orleans, LA, United states

[19] Elisseeff A, Weston J (2002) A kernel method for multi-labelled classification. Adv Neural Inf Process Syst 681–687

[20] Fan M, Gu N, Qiao H, Zhang B (2014). Dimensionality reduction: an interpretation from manifold regularization perspective. Inf Sci.

[21] Feng S, Wang Y, Song K, Wang D, Yu G (2018). Detecting multiple coexisting emotions in microblogs with convolutional neural networks. Cogn Comput.

[22] Friedman M (1940). A comparison of alternative tests of significance for the problem of m rankings. Ann Math Stat.

[23] Garcia S, Herrera F (2008). An extension on“statistical comparisons of classifiers over multiple data sets” for all pairwise comparisons. J Mach Learn Res.

[24] Granville A (1989) On elementary proofs of the prime number theorem for arithmetic progressions, without characters. In: Proceedings of the Amalfi Conference on Analytic Number Theory, pp 157–195

[25] Hu W, Gao J, Xing J, Zhang C, Maybank S (2017). Semi-supervised tensor-based graph embedding learning and its application to visual discriminant tracking. IEEE Trans Pattern Anal Mach Intell.

[26] Jebara T, Wang J, Chang SF (2009) Graph construction and b-matching for semi-supervised learning. In: Proceedings of the 26th Annual International Conference on Machine Learning, pp 441–448. ACM

[27] Ji M, Zhang K, Wu Q, Deng Z (2020). Multi-label learning for crop leaf diseases recognition and severity estimation based on convolutional neural networks. Soft Comput.

[28] Joachims T (2003) Transductive learning via spectral graph partitioning. In: Proceedings of the 20th International Conference on Machine Learning (ICML-03), pp 290–297

[29] Li D, Dick S (2019). Residential household non-intrusive load monitoring via graph-based multi-label semi-supervised learning. IEEE Trans Smart Grid.

[30] Li D, Dick S (2017) A graph-based semi-supervised learning approach towards household energy disaggregation. In: Fuzzy Systems (FUZZ-IEEE), 2017 IEEE International Conference on, pp 1–7. IEEE, Naples, Italy

[31] Madjarov G, Kocev D, Gjorgjevikj D, Džeroski S (2012). An extensive experimental comparison of methods for multi-label learning. Pattern Recogn.

[32] Mallapragada PK, Jin R, Jain AK, Liu Y (2009). Semiboost: boosting for semi-supervised learning. IEEE Trans Pattern Anal Mach Intell.

[33] Mehrkanoon S, Suykens JA (2016) Multi-label semi-supervised learning using regularized kernel spectral clustering. In: Neural Networks (IJCNN), 2016 International Joint Conference on, pp 4009–4016. IEEE

[34] MEŠTROVIC R (2012) Euclid’s theorem on the infinitude of primes: a historical survey of its proffs (300 bc–2012) and another new proof. arXiv preprint arXiv:1202.3670

[35] Minh HQ, Sindhwani V (2011) Vector-valued manifold regularization. Int Conf Mach Learn 57–64

[36] Minh HQ, Bazzani L, Murino V (2016). A unifying framework in vector-valued reproducing kernel hilbert spaces for manifold regularization and co-regularized multi-view learning. J Mach Learn Res.

[37] Moyano JM, Gibaja EL, Cios KJ, Ventura S (2018). Review of ensembles of multi-label classifiers: models, experimental study and prospects. Inf Fusion.

[38] Narkiewicz W (2013). The development of prime number theory: from Euclid to Hardy and Littlewood.

[39] Rivolli A, Read J, Soares C, Pfahringer B, de Carvalho ACPLF (2020). An empirical analysis of binary transformation strategies and base algorithms for multi-label learning. Mach Learn.

[40] Schölkopf B, Herbrich R, Smola AJ (2001) A generalized representer theorem. In: International Conference on Computational Learning Theory, pp 416–426. Springer

[41] Scudder H (1965). Probability of error of some adaptive pattern-recognition machines. IEEE Trans Inf Theory.

[42] Seeger, M.: Learning with labeled and unlabeled data. Tech. rep., Institute for Adaptive and Neural Computation, University of Edinburgh (2000)

[43] Selberg A (1949) An elementary proof of Dirichlet’s theorem about primes in an arithmetic progression. Ann Math 297–304

[44] Sheldon MR, Fillyaw MJ, Thompson WD (1996). The use and interpretation of the Friedman test in the analysis of ordinal-scale data in repeated measures designs. Physiother Res Int.

[45] Sindhwani V, Keerthi SS, Chapelle O (2006) Deterministic annealing for semi-supervised kernel machines. In: Proceedings of the 23rd International Conference on Machine Learning, pp 841–848. ACM

[46] Snoek CG, Worring M, Van Gemert JC, Geusebroek JM, Smeulders AW (2006) The challenge problem for automated detection of 101 semantic concepts in multimedia. In: Proceedings of the 14th ACM International Conference on Multimedia, pp 421–430. ACM

[47] Sorower MS (2010) A literature survey on algorithms for multi-label learning. Tech. rep., Oregon State University, Corvallis

[48] Subramanya A, Bilmes J (2011). Semi-supervised learning with measure propagation. J Mach Learn Res.

[49] Subramanya A, Talukdar PP (2014) Graph-based semi-supervised learning. Synthesis Lectures on Artificial Intelligence and Machine Learning 8(4):1–125

[50] Sun S, Xie X (2016). Semisupervised support vector machines with tangent space intrinsic manifold regularization. IEEE Trans Neural Netw Learn Syst.

[51] Szummer M, Jaakkola T (2002) Partially labeled classification with Markov random walks. Adv Neural Inf Process Syst 945–952

[52] Trohidis K, Tsoumakas G, Kalliris G, Vlahavas IP (2008). Multi-label classification of music into emotions. ISMIR.

[53] Tu E, Zhang Y, Zhu L, Yang J, Kasabov N (2016). A graph-based semi-supervised k nearest-neighbor method for nonlinear manifold distributed data classification. Inf Sci.

[54] Vapnik V (1998). Statistical learning theory.

[55] Wang J, Jebara T, Chang SF (2013). Semi-supervised learning using greedy max-cut. J Mach Learn Res.

[56] Zha ZJ, Mei T, Wang J, Wang Z, Hua XS (2009). Graph-based semi-supervised learning with multiple labels. J Vis Commun Image Represent.

[57] Zhang ML, Zhou ZH (2007). ML-KNN: a lazy learning approach to multi-label learning. Pattern Recogn.

[58] Zhang ML, Zhou ZH (2014). A review on multi-label learning algorithms. IEEE Trans Knowl Data Eng.

[59] Zhang Z, Zhao M, Chow TW (2015). Graph based constrained semi-supervised learning framework via label propagation over adaptive neighborhood. IEEE Trans Knowl Data Eng.

[60] Zhao Y, Zhao Y, Zhu Z (2009). TSVM-HMM: Transductive SVM based hidden Markov model for automatic image annotation. Expert Syst Appl.

[61] Zhao Y, Ball R, Mosesian J, de Palma JF, Lehman B (2015). Graph-based semi-supervised learning for fault detection and classification in solar photovoltaic arrays. IEEE Trans Power Electron.

[62] Zhou ZH, Li M (2010). Semi-supervised learning by disagreement. Knowl Inf Syst.

[63] Zhou X, Belkin M (2013) Semi-supervised learning. In: Chapter 22, Academic Press Library in Signal Processing

[64] Zhou D, Bousquet O, Lal TN, Weston J, Schölkopf B (2004) Learning with local and global consistency. Adv Neural Inf Process Syst 321–328

[65] Zhu X (2005) Semi-supervised learning literature survey. Tech. Rep. 1530, Computer Sciences, University of Wisconsin-Madison

[66] Zhu X, Ghahramani Z (2002) Learning from labeled and unlabeled data with label propagation. In: Technical Report CMU-CALD-02-107. Citeseer

[67] Zhu X, Ghahramani Z, Lafferty JD (2003) Semi-supervised learning using gaussian fields and harmonic functions. In: Proceedings of the 20th International conference on Machine learning (ICML-03), pp 912–919

[68] Zhu X, Goldberg AB (2009) Introduction to semi-supervised learning. Synthesis lectures on artificial intelligence and machine learning 3(1):1–130

